# Reintroduction Without Genetic Bottlenecks: Preserving Diversity in Restored Populations of the Critically Endangered Riverine Shrub *Myricaria germanica*

**DOI:** 10.1002/ece3.72879

**Published:** 2026-01-11

**Authors:** Katerina Iberl, Christoph Reisch

**Affiliations:** ^1^ Department of Botany Charles University Prague Czech Republic; ^2^ Institute of Plant Sciences University of Regensburg Regensburg Germany

**Keywords:** Alpine rivers, founder and bottleneck effects, genetic variation, hydrological alterations, *Myricaria germanica*, restoration measures

## Abstract

River regulation has profoundly altered hydrological regimes in alpine floodplains, leading to severe habitat loss and population declines of specialist species such as *Myricaria germanica*. Since the early 2000s, extensive reintroduction programs have been implemented to counteract the consequences of channelization and embankment, aiming to prevent the extinction of this characteristic gravel‐bank shrub. Yet, the genetic outcomes of these measures remain largely unexplored, while the potential influence of founder and bottleneck effects on restoration success remains uncertain. We analyzed genetic variation within and among natural and restored populations along the rivers Isar and Lech in southern Germany and northern Austria. Restored populations exhibited genetic diversity comparable to natural populations along the Isar, and differentiation between seed source and restored sites was modest (PhiPT = 0.12). Our findings show that restoration relying on a single, genetically variable source population can successfully maintain local genetic diversity. This highlights that targeted reintroduction can safeguard genetic variation while avoiding possible long‐term risks of excessive mixing, such as potential outbreeding depression or reduced fitness, thereby supporting the persistence of this iconic riverine species under ongoing environmental change.

## Graphical abstract

Anthropogenic river regulation has severely reduced habitats of alpine floodplain specialists such as Myricaria germanica. Analyses of natural and restored populations along the Isar and Lech Rivers revealed that reintroduction from a single, genetically variable source can maintain local genetic diversity, with only modest differentiation from the seed source. These findings demonstrate that targeted restoration based on a well‐chosen source population can safeguard local genetic variation while avoiding uncertain long‐term risks of excessive mixing, thereby supporting the persistence of this iconic species in the face of changing environmental conditions.

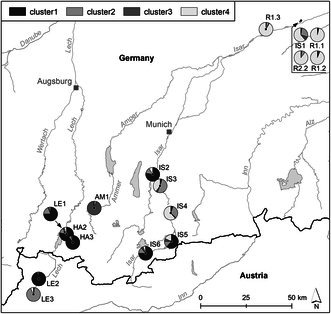

## 1 Introduction

Ecological restoration is a powerful tool to counteract the decline of natural habitats and biodiversity, including the hidden and irreversible erosion of genetic diversity. One of the most important instruments in restoration is species reintroduction (IUCN/SSC 2013) aimed at establishing viable, self‐sustaining populations that reproduce independently and persist across future decades. However, reintroduction is a complex process, and its success depends on several key factors. Among these, the genetic diversity of both the seed source and the restored populations is fundamental for evolutionary resilience and long‐term viability (Drayton and Primack 2012; Kettenring et al. 2014; Bucharova et al. 2019; Kyriazis et al. 2021).

Before restoration efforts begin, one or more suitable source populations must be identified and selected. This is an important step, as limited genetic diversity in the source population may result in low establishment rates and reduced performance of restored populations (Godefroid et al. 2011; Basey et al. 2015). Especially small and isolated populations are prone to reduced genetic diversity and the effects of genetic drift (Leimu and Fischer 2008; Dostálek et al. 2010; Busch and Reisch 2015). In contrast, present or past connectivity can foster genetic diversity within populations and mitigate genetic differentiation among them (Delnevo et al. 2021).

Moreover, establishing new populations from a limited number of individuals may foster inbreeding and reduce performance in restored populations (Robichaux et al. 1997; Godefroid et al. 2011; Taylor et al. 2017), thus undermining their adaptive potential under changing environmental conditions (Brown and Briggs 1991; Frankham et al. 2010; Bucharova et al. 2019).

Therefore, Franklin (1980) and Soulé and Wilcox (1980) proposed the much‐discussed 50/500 rule, according to which 50 individuals should avoid inbreeding depression, while 500 individuals should maintain evolutionary potential. For seed sourcing, Brown and Briggs (1991) suggest collecting from at least 50–100 randomly chosen individuals per population to ensure sufficient genetic representation. Increasing sample size is generally beneficial, particularly for ensuring long‐term viability. This approach allows recovery of at least one copy of alleles with frequencies above 0.05–0.10, with a probability of approximately 90%–95%.

Sourcing seeds from multiple populations can enhance genetic diversity and increase the viability of restored populations (Vergeer et al. 2005; Guerrant Jr and Kaye 2007; Gabel et al. 2017; St. Clair et al. 2020) particularly under changing environmental conditions. This strategy often leads to greater reintroduction success (Godefroid et al. 2011) and is widely used in large‐scale restoration projects (Havens et al. 2015; Höfner et al. 2022). However, this approach may be counterproductive when the aim is to preserve a distinct population structure. In such cases, sourcing seeds from a single population or neighboring subpopulations is more appropriate (Betz et al. 2013; Kaulfuß and Reisch 2017). Moreover, for species with distinct population structures, mixing sources may lead to outbreeding depression and reduced offspring fitness (Montalvo and Ellstrand 2001; Rodger et al. 2024; Bürli et al. 2025).

The spatial scale of seed sourcing should reflect the natural or historical extent of gene flow in the target species. This approach helps preserve genetic structure and prevents unintended consequences such as outbreeding depression. Ideally, this range is determined through prior genetic analyses. For example, Durka et al. (2017) demonstrated pronounced genetic differentiation in grassland species across Germany, supporting the importance of regionally adapted seed transfer zones for ecological restoration.

In the context of critically endangered species, genetic analyses provide a means to identify populations of highest value for the conservation of allele diversity. These populations may serve as seed sources for reintroduction efforts (Iberl et al. 2024). Highly specialized or isolated populations—for example, endemics restricted to glacial refugia or mountain summits—may harbor unique genetic adaptations or allelic combinations absent elsewhere (Záveská et al. 2021). In such cases, reinforcement or reintroduction should draw exclusively on material from the same population to prevent genetic dilution and safeguard locally adapted traits.

In our study, we examined how reintroduction influences genetic variation in the endangered plant *Myricaria germanica* Desv. (Tamaricaceae). This species is a long‐lived shrub (10–20 years) adapted to dynamic riverine landscapes with alternating dry and moist regimes. It naturally occurs on sandy and gravelly banks of alpine rivers and also colonizes secondary habitats such as gravel pits, quarries, and railway embankments (Marinov et al. 2017). The species is distributed from Central Europe to Central Asia, the presumed center of origin of its genus (Sun and Werth 2024). Its establishment critically depends on regular flooding and open, nutrient‐poor substrates, as shown by studies on its phenology and habitat preferences (Lener et al. 2013; Egger et al. 2017). *Consequently, M. germanica* represents a specialist and a key indicator of intact, dynamic floodplain ecosystems (Kudrnovsky 2013). Decades of anthropogenic river regulation—such as damming and bank stabilization—have profoundly altered natural river dynamics. This has resulted in a substantial loss of suitable habitats and a marked decline in population density (Sitzia et al. 2021). In particular, reduced flood frequency hampers regeneration and limits the species' capacity to compete (Gostner et al. 2017).

*M. germanica* is a diploid species (2n = 24; Váchová and Májovský 1978) that exhibits a mixed mating system characterized by frequent selfing (Werth and Scheidegger 2014). The species flowers from June to August in terminal or lateral racemes, relying on generalist insects for pollination (Tutin 1968). Seeds germinate in light and disperse via wind and water, aided by their buoyant structure (Bill 2000). Studies of population dynamics (Sitzia et al. 2016) and colonization potential (Fink et al. 2017) reveal that the species has a certain dispersal capacity, yet its establishment remains highly dependent on specific site conditions. Metapopulation research along the River Isel (Austria) revealed bidirectional gene flow, with first‐generation migrants detected in both tributaries and the main channel, confirming upstream dispersal via seeds and pollen. Tributaries may therefore function as refugia during extreme flood events (Fink et al. 2022).

Previous genetic studies revealed a partially pronounced population structure and regional differentiation (Wiedmer and Scheidegger 2014; Werth and Scheidegger 2014; Werth et al. 2014), thereby implicitly supporting the use of local provenances in restoration efforts. While insights from intact alpine systems provide valuable guidance, their applicability to the highly fragmented edge populations outside the Alps included in this study remains uncertain. Our study addresses this issue by testing whether strict reliance on local single‐source material can function effectively in such contexts.

At the same time, numerous well‐documented reintroduction efforts have been undertaken to prevent extinction in recent decades. Such initiatives have been reported from Austria (Latzin and Schratt‐Ehrendorfer 2005; Kudrnovsky 2013), Italy (Michielon and Sitzia 2015), and Germany (Harzer et al. 2018; Egger et al. 2010; Riehl and Zehm 2018), yet their genetic impact remains largely unknown. To bridge this gap, we analyzed genetic variation in natural and restored populations along the River Isar and compared them with populations from the River Lech, thereby providing a broader perspective on the species' genetic diversity and structure at the periphery of its distribution in Central Europe.

More specifically, we asked the following questions: (i) Are there differences between the restored and natural populations in terms of genetic diversity? Is there evidence of genetic differentiation between restored and natural populations? (ii) How high is the genetic diversity within and the genetic differentiation between natural populations of *M. germanica* along the Isar and Lech rivers? (iii) What conclusions can be drawn for the current reintroduction and the seed sourcing strategy for future restoration measures?

## 2 Materials and Methods

### 2.1 Study Design and Sampled Populations

We investigated 16 populations in total, of which 12 were natural, three restored and one offspring population descending from one of the restored populations (Table 1). The natural populations comprised (i) six populations growing along the river Isar, (ii) five populations along the river Lech and (iii) one remaining natural population situated at the river Ammer (Table 1). One of the natural Isar populations (IS1) was used as a seed source for restoration. We investigated three restored populations originating from IS1. One of them (R1.2) produced offspring, which spontaneously emerged in its vicinity. We enclosed this offspring population (R2.2) into the study. If available, 16 plants per population were sampled. Where feasible, populations were sampled along transects to maximize genetic representativeness. In IS2, a subpopulation on a river island was included. Sampling in IS6 was stratified across three distinct sections to capture intra‐population variation. Terminal branches lacking buds or flowers were collected, wrapped in little paper bags (teabags) and immediately dried over silica gel. Dried plant material was stored in boxes containing an active silica gel until the DNA extraction.

**TABLE 1:** Population information for individuals of the critically endangered shrub *Myricaria germanica*.

Nr	Name	Abb.	Region	N/R	PS	Altitude	*n*
1	Helfenwang	LE1	Swabia	N	1000	750	15
2	Forchach	LE2	Tyrol	N	100	900	16
3	Martinau	LE3	Tyrol	N	30	960	14
4	Eschenberg	HA2	Swabia	N	300	760	15
5	Im Laich	HA3	Swabia	N	50	890	15
6	Rosenau	IS1	Lower Bavaria	N	5	350	5
7	Puppling	IS2	Upper Bavaria	N	200	570	15
8	Geretsried	IS3	Upper Bavaria	N	300	610	16
9	Gaissach	IS4	Upper Bavaria	N	50	650	16
10	Sylvenstein	IS5	Upper Bavaria	N	100	710	14
11	Ochsensitz	IS6	Upper Bavaria	N	> 2000	810	16
12	Kohlbach	AM1	Upper Bavaria	N	15	620	13
13	Rosenau	R1.1	Lower Bavaria	R	—	350	16
14	Rosenau	R1.2	Lower Bavaria	R	—	350	16
15	Postau	R1.3	Lower Bavaria	R	—	380	13
16	Rosenau	R2.2	Lower Bavaria	R	—	350	15

*Note:* The table lists the population number (Nr), name, abbreviation (Abb.), region (SW, Swabia; TIR, Tyrol; LB, Lower Bavaria; UB, Upper Bavaria), population type (*N*, natural, *R*, restored), estimated population size (PS), altitude (m above sea level), and the number of individuals analyzed (*n*).

Restoration was conducted along the lower course of the Isar River, using seeds from the last surviving natural population in that area. Despite its small size and irregular reproduction, it was chosen to preserve local genetic purity (Bischoff et al. 2006; Mijnsbrugge et al. 2010). Mature diaspores were collected over several years and sown immediately. Seedlings were raised in a local nursery for 2 years before being planted at restoration sites. The R1.1 and R1.2 populations were located near the natural seed source IS1, while RE1.3 was planted 20 km from the source.

### 2.2 Molecular Analyses

We analyzed a total of 228 plants, 14–16 individuals per population. We aimed to achieve as similar a number of samples per population as possible. Leipold et al. (2020) demonstrated that even 12–13 samples per population are sufficient to capture 99% of alleles. In populations consisting of a lower number of plants, we confined our analysis to the number of available individuals. In the larger populations, samples were selected randomly across the populations.

In our study, we used AFLP markers because they offered practical benefits that justified their application. As a well‐established method in conservation genetics, AFLPs provide a cost‐effective and time‐efficient means of screening genome‐wide variation across many individuals and populations. The protocol is routinely applied in our laboratory with proven reproducibility. Although AFLPs are dominant markers and do not allow direct heterozygosity estimates, they are well suited for assessing genetic structure, differentiation, and assignment when combined with frameworks such as STRUCTURE, AMOVA, and AFLPop. Both AMOVA and STRUCTURE have been extensively validated for AFLP data, and AFLPop specifically accounts for the dominant nature of the markers, enabling reliable individual assignment in ecological and conservation contexts.

Genomic DNA for AFLPs was isolated from dried plant material following the CTAB protocol (Rogers and Bendich 1994), adapted as described in previous studies (Reisch et al. 2005). Concentration of DNA stock solutions was diluted with water to 7.8 ng/μL and used for AFLP analyses. The AFLP analyses were conducted according to the protocol from Beckmann Coulter, including slight modifications as described in former studies (Reisch 2007; Bylebyl et al. 2008). Preselective amplification used primers with one selective nucleotide (MseI‐C and EcoRI‐A; Vos et al. 1995).

Selective amplification was carried out using three primer combinations MseI‐CAA/EcoRI‐AAC (D2), MseI‐CTA/EcoRI‐AAG (D3), MseI‐CAT/EcoRI‐ACA (D4). We selected these three primer combinations based on prior screening of multiple candidates with three selective nucleotides. This combination produced clear, distinct, and reproducible banding patterns in the AFLP workflow. The amplification products were diluted as follows: 2‐fold (D2) and 5‐fold (D4) using TE0.1 buffer for DNA. The fluorescence‐labeled samples were separated by capillary gel electrophoresis on an automated sequencer (GenomLab GeXP, Beckman Coulter). Raw data were examined applying the GeXP software (Beckman Coulter) and analyzed using the software Bionumerics 4.6 (Applied Maths). Across all samples, each band was scored as either present or absent.

To assess reproducibility of molecular analyses, we estimated the genotyping error rate following Bonin et al. (2004). Approximately 10% of all samples were randomly selected and analyzed twice using AFLPs. The resulting binary matrices were compared across all loci, and differences in band presence/absence were recorded as mismatches. In total, 2540 loci were scored, of which 116 showed inconsistencies, corresponding to an overall error rate of 4.57%.

### 2.3 AFLP Statistics

Based on the AFLP data, a binary matrix was generated using Bionumerics 4.6 (Applied Maths). Bands of a given length were scored as present (1) or absent (0). From this 0/1 matrix, the percentage of polymorphic bands across the dataset was calculated as the ratio *n*_*i*_/*N*, where *n*_*i*_ is the number of polymorphic bands and *N* the total number of fragments. To reduce statistical bias, all monomorphic bands across individuals were excluded from subsequent analyses (Keiper and McConchie 2000; Li et al. 2005). Genetic diversity within populations was calculated with AFLPsurv (Vekemans 2002). Allelic frequencies were estimated using a Bayesian approach with a nonuniform prior distribution (Zhivotovsky 1999). Nei's gene diversity (*H*) was employed as a measure of genetic diversity, following Lynch and Milligan (1994) and computed as *H* = 1 − Σ(*p*_*i*_)^2^ where *p*_*i*_ represents allele frequency (Nei 1973). The impact of population size on genetic diversity indices was tested using Pearson's correlation coefficient, with population size log‐transformed on the x‐axis.

Differences in genetic diversity between natural populations along the Isar River and restored populations were tested using a *t*‐test implemented in R (version 2024.12.0.467; Posit Team 2024), following a prior assessment of normality with the Shapiro–Wilk test.

The *t*‐test was performed only for PPL and Rarity, as these metrics showed some variation between catchments. All other metrics were highly similar across groups, and therefore the *t*‐test was considered redundant and omitted.

Genetic distances among populations were calculated as Nei's standard distance (Ds) with a nonuniform prior distribution of allele frequencies using AFLP‐surv (Vekemans 2002). Based on these distances, a consensus Neighbor‐Net graph was constructed with SplitsTree4 (Huson and Bryant 2006).

Analyses of molecular variance (AMOVA) based on pairwise Euclidean distances between samples were conducted in GenAlex 6.41 (Peakall and Smouse 2006), with significance levels assessed using 999 permutations. Differentiation was tested between the two river catchments, among populations within catchments, and within populations. Pairwise *Φ*_PT_ values were calculated for all populations. To evaluate isolation by distance (IBD), Mantel tests were performed by correlating pairwise genetic distances (*Φ*_PT_) with geographical distances among populations (Mantel 1967). The impact of population size on genetic diversity was further assessed using Pearson's correlation coefficient, implemented in R with the ggplot2 package (Posit team 2024).

To visualize genetic similarities among individuals, a Principal Coordinates Analysis (PCoA) was performed. A Jaccard distance matrix was first calculated based on the binary AFLP data using the vegdist function from the vegan package. Dimensionality reduction was then carried out via classical multidimensional scaling (cmdscale), extracting the first two principal coordinates. The resulting PCoA coordinates were visualized using the ggplot2 package. Individuals were color‐coded according to their population assignment, with custom colors manually defined to maximize visual contrast and clarity. Axis labels indicate the percentage of total variance explained by each principal coordinate.

The genetic structure of populations was inferred using Bayesian cluster analysis implemented in STRUCTURE 2.3.4 (Pritchard et al. 2000, 2009). Population structure was assessed by assigning individuals to groups. For datasets with an unknown number of clusters (*K*), 500,000 Markov Chain Monte Carlo iterations were performed with a burn‐in of 250,000 to identify the most likely number of groups. Analyses for each predefined value of *K* (ranging from 1 to 16) were replicated 20 times (Falush et al. 2007). We used Structure Harvester (Earl and von Holdt 2012) to summarize the results. Analyses were conducted under the admixture ancestry model with correlated allele frequencies, which is appropriate for species with mixed reproductive systems involving both outcrossing and gene flow among populations. The optimal number of clusters was determined at the point where the mean logarithm of the probability of the data [Ln *p*(X|*K*)] approached a plateau, such that further increases in *K* provided little additional information (Pritchard et al. 2000).

To assess gene flow among the studied populations and potential immigration events from outside, we employed AFLPOP 1.2 (Duchesne and Bernatchez 2002). Assignment tests implemented in the program identified the most probable source population for each individual. Analyses were conducted using the allocation procedure of unknown origin, with marker frequencies of zero replaced according to the formula [1/(sample size +1)]. Individuals were allocated at three levels, applying different minimal log‐likelihood differences (MLDs; Raffl et al. 2006; Pollux et al. 2009). AFLPOP assigns an individual to a source population X if its likelihood within X is at least 10^MLD times higher than that of the next most likely population (Duchesne and Bernatchez 2002). MLDs were set at 0.5 (allocation only if likelihood was ≥ 3 times higher), 0.3 (≥ 2 times higher), and 0 (allocation to the most probable source population). Correctly assigned individuals (CA) were allocated to their population of origin, mismatched assignments (MA) indicated origin from another study population, and not assigned individuals (NA) were most likely from populations not included in the study. The number of simulated genotypes for calculating *p* values was set to 500.

A short terminology remark is included to clarify the use of diversity‐related terms: In this study, we use the term *genetic variation* as an umbrella concept including both within‐population and between‐population differences. *Genetic diversity*, by contrast, refers specifically to variation within populations, as captured by indices such as the percentage of polymorphic loci (PPL) and rarity. To ensure clarity, we consistently refer to specific metrics when reporting results.

## 3 Results

### 3.1 Genetic Diversity

AFLP genotyping of 229 plants resulted in 209 fragments (CAA‐AAC: 67 fragments, CTA‐AAG: 71 fragments, CAT‐ACA: 71 fragments), of which 44.02% were polymorphic. Genetic diversity indices were similar across natural and restored populations, with no significant differences detected. Slightly higher values were observed in restored populations for most indices except Nei's gene diversity (Table 2). The highest diversity was found in LE1, IS3, and AM1, and RE2.2 among restored populations. A *t*‐test comparing the percentage of polymorphic loci (PPL) between Isar (mean = 18.82%) and Lech (mean = 23.74%) showed no statistically significant difference.

**TABLE 2:** Indicators of genetic diversity measured across populations of *Myricaria germanica*.

Nr	Abbreviation	Ne	*H*	SI	PPL	Rarity (DW)
	**Natural Lech**					
1	LE1	1.25	0.20	0.22	41.12	0.43
2	LE2	1.10	0.12	0.09	18.69	0.53
3	LE3	1.13	0.11	0.11	21.50	0.40
4	HA2	1.13	0.09	0.11	18.69	0.42
5	HA3	1.12	0.12	0.10	18.69	0.45
	**Mean Natural Lech**	1.15 ± 0.06	0.13 ± 0.04	0.13 ± 0.05	23.74 ± 9.79	0.45 ± 0.05
	**Natural Isar**					
6	IS1	1.16	0.19	0.12	17.76	0.44
7	IS2	1.11	0.10	0.09	16.82	0.43
8	IS3	1.18	0.16	0.14	23.36	0.45
9	IS4	1.12	0.11	0.10	17.76	0.40
10	IS5	1.11	0.11	0.10	18.69	0.48
11	IS6	1.15	0.13	0.12	22.43	0.50
12	AM1	1.08	0.07	0.07	14.95	0.71
	**Mean Natural Isar**	1.13 ± 0.03	0.13 ± 0.03	0.11 ± 0.04	18.82 ± 3.02	0.49 ± 0.10
	**Mean Natural Lech & Isar**	1.14 ± 0.04	0.13 ± 0.04	0.11 ± 0.04	20.87 ± 6.80	0.47 ± 0.08
	**Restored Isar**					
13	R1.1	1.10	0.09	0.09	15.89	0.46
14	R1.2	1.10	0.09	0.08	15.89	0.48
15	R1.3	1.16	0.14	0.12	18.69	0.43
16	R2.2	1.30	0.17	0.25	42.06	0.46
	**Mean Restored**	1.17 ± 0.09	0.11 ± 0.02	0.14 ± 0.08	23.13 ± 12.69	0.46 ± 0.02
***T*‐test (Natural Isar and Restored Isar)**		*p* value = 0.39	*p* value = 0.94	*p* value = 0.37	*p* value = 0.40	*p* value = 0.59

*Note:* For all measures, mean values ± standard deviation was given. Further, results of the *t*‐tests are shown (*p*‐values); “±” refers to standard deviation.

Abbreviations: DW, the Frequency‐down‐weighted marker value; *H*, unbiased Nei's Gene Diversity; Ne, effective number of alleles; PPL, percentage of polymorphic loci; SI, Shannon's Information Index.

No significant correlation was found between population size and overall genetic diversity. However, a marginally significant relationship emerged for one specific measure—the percentage of polymorphic loci PPL (*p* = 0.055)—with a Pearson coefficient of *R* = 0.57, indicating a moderate correlation between these variables (Figure S1).

We also assessed the occurrence and distribution of private and rare fragments (Tables S1 and S2). The highest number of private fragments (*n* = 3) was found in population AM1, while IS6 and LE2 each contained one. Rare fragments (frequency < 5%) were present in both the Isar (IS2, IS5, AM1) and Lech catchments (LE2, HA1, HA2). Additionally, rare fragments were detected in three of the four restored populations (R1.1, R1.2, R2.2).

### 3.2 Genetic Differentiation, Structure and Gene Flow Among Populations

Genetic differentiation among all natural populations was *Φ*_PT_ = 0.31. Within the Isar populations (excluding its tributary, the Ammer River), differentiation was *Φ*_PT_ = 0.21, while among the Lech and Halblech populations it was *Φ*_PT_ = 0.29. The three‐level AMOVA revealed 4% differentiation between the Isar and Lech catchments (Table 3). The difference between the natural seed‐source population IS1 and the restored populations was modest (8%) and nonsignificant, whereas genetic differentiation among the restored populations was 4% (*p* = 0.03). No significant differentiation was detected between the natural offspring population R2.2 and the natural seed‐source population IS1 (Table S3).

**TABLE 3:** Molecular variance among populations of *Myricaria germanica* based on AMOVA.

Source of variation	df	SS	MS	%	*Φ*_PT_
All natural populations					
Among populations	11	269.5	24.5	31	0.31***
Within populations	158	518.1	3.3	69	
Isar (without Ammer)					
Among populations	5	79.5	15.9	21	0.21***
Within populations	76	267.8	3.5	79	
Lech					
Among populations	4	94.9	23.7	29	0.29***
Within populations	70	232.3	3.3	71	
Lech and Isar					
Between the Lech and the Isar River	1	36.8	36.8	4	0.32***
Among populations within the groups	10	232.8	23.3	24	
Within populations	158	518.1	3.2	72	
Restored and the natural population IS1					
Among groups	1	6.3	6.3	8	0.12*
Among populations	2	9.8	4.9	4	
Within populations	46	137.1	2.9	88	

*Note:* Levels of significance are based on 999 iteration steps and are indicated by three (*p* < 0.001), or one (*p* < 0.05) asterisk.

Abbreviations: %, the proportion of genetic variation; df, degree of freedom; MS, the mean squares; SS, the sum of squares; *Φ*_PT_, the level of genetic differentiation.

Among the natural populations, correlations between pairwise genetic distances (*Φ*_PT_) and geographic distances were not significant. Both direct‐line and along‐river distances were considered, and analyses were conducted for the Isar and Lech catchments jointly as well as separately. Within the Lech catchment, no significant correlation was detected (Mantel test: *R* ^2^ = 0.0975, *p* = 0.18). In contrast, within the Isar catchment we observed a marginally significant association between genetic and geographic distances (Mantel test: *R* ^2^ = 0.5062, *p* = 0.058).

**FIGURE 1:**
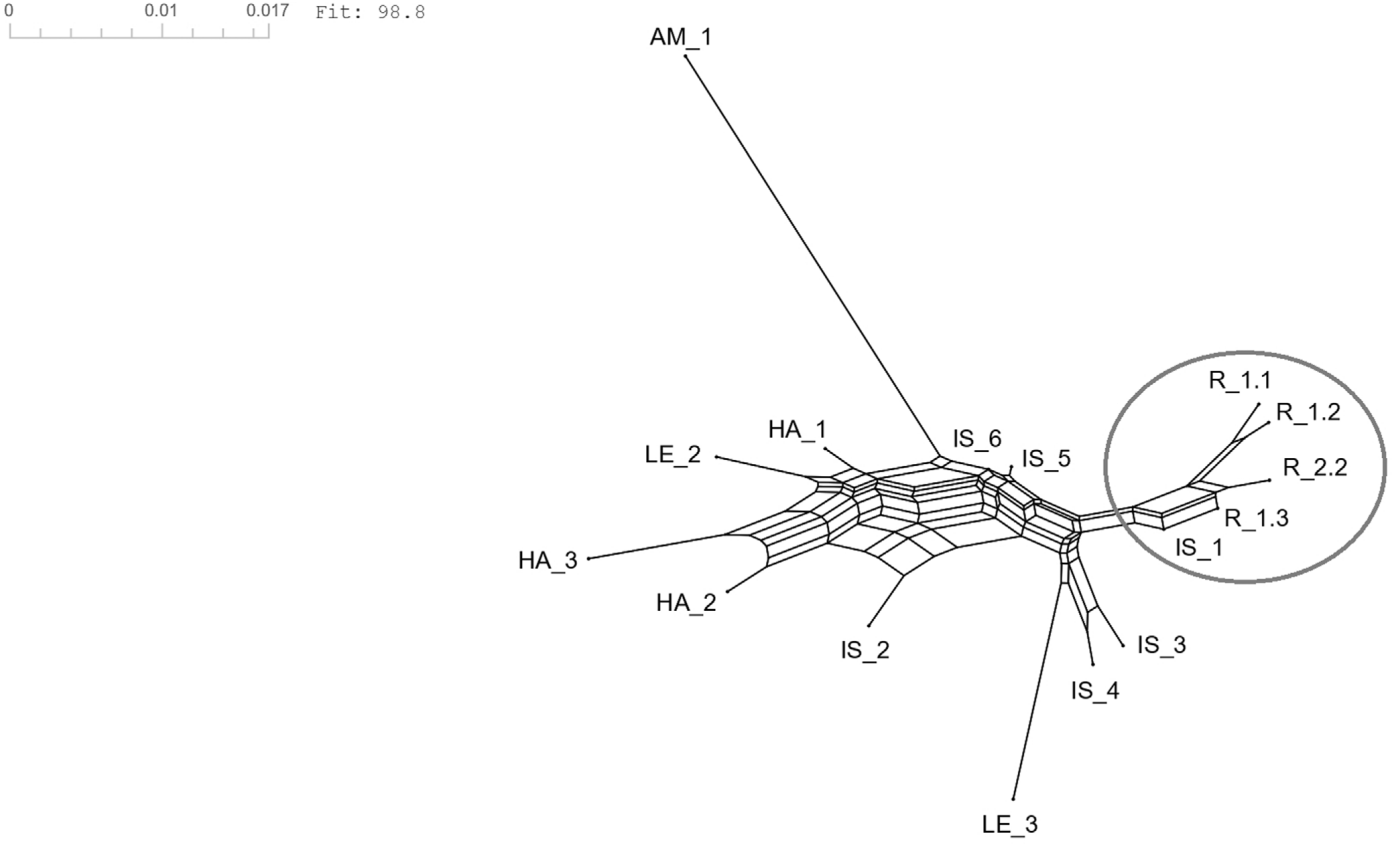
Neighbor‐net network based on Nei's genetic distances illustrating the genetic relationships among all studied *Myricaria germanica* populations. The natural seed‐source population IS1 clusters closely with the restored populations R1.1, R1.2, and R1.3, as well as with the natural offspring population R2.2. These genetically associated populations are visually emphasized by a circle in the right portion of the network.

The Neighbor‐Net analysis revealed that the natural seed‐source population IS1, the restored populations R1.1, R1.2, R1.3, and the natural offspring population R2.2 formed a consistent cluster, located on the right side of the graph (Figure 1). The remaining populations did not reflect geographic configuration, such as proximity or affiliation with a particular river catchment. Only the Ammer population (AM1) formed a distinct branch, clearly separated from all other populations.

**FIGURE 2:**
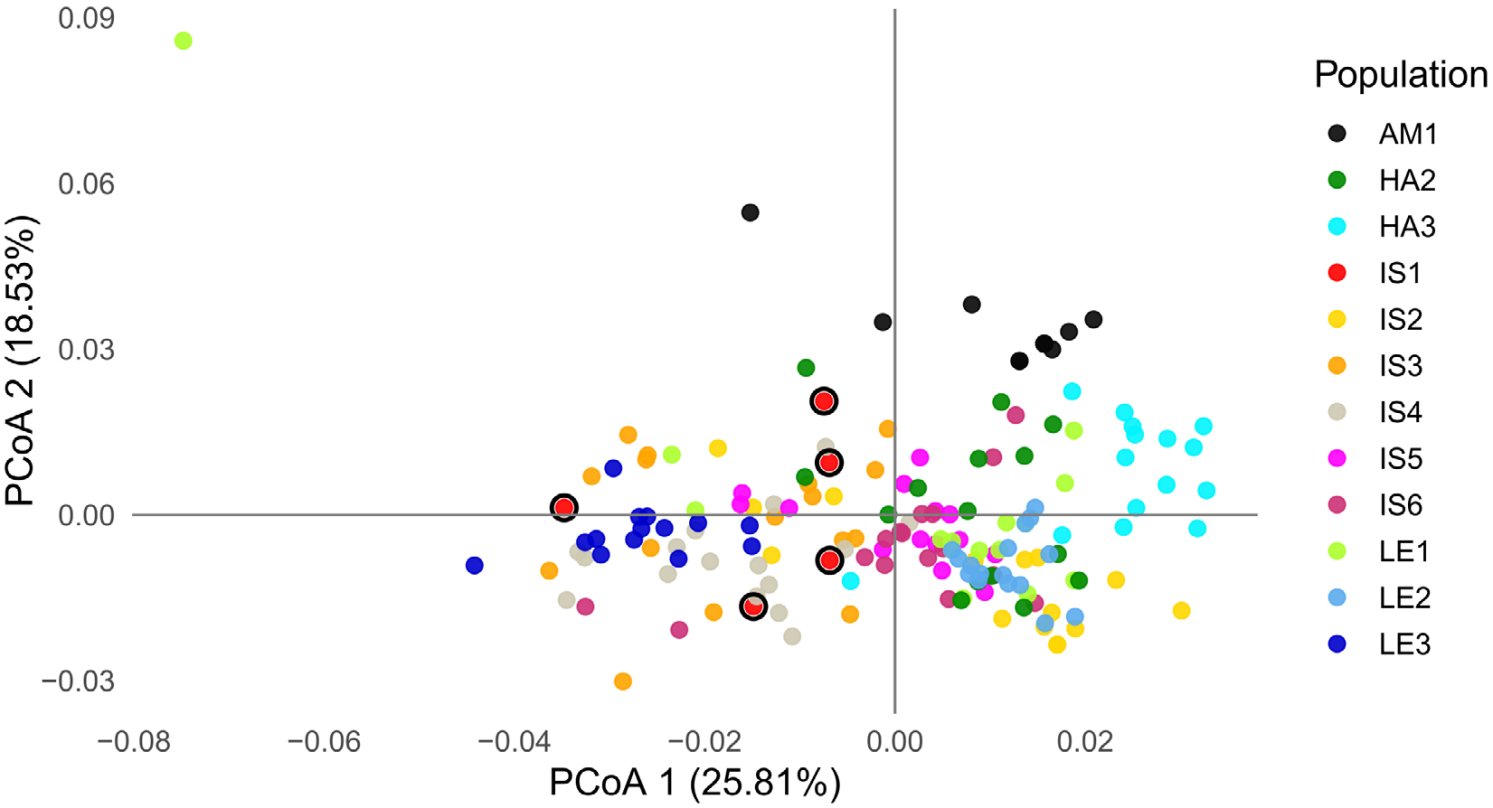
Principal coordinates analysis (PCoA) of 170 individuals from natural populations of *Myricaria germanica*, based on Jaccard distances. The first and second axes account for 25.81% and 18.53% of the total genetic variation, respectively. Populations show limited separation in the ordination space, indicating ongoing/historical gene flow and dispersal within and between the Isar and Lech River catchments. The seed‐source population IS1 is marked by a red filled point encircled in black.

Principal coordinates analysis (PCoA) of 170 individuals from natural populations of xy, based on Jaccard distances, revealed that the first and second axes accounted for 25.81% and 18.53% of the total genetic variation, respectively. The ordination did not reveal a clear separation among most populations, indicating substantial gene flow and dispersal within and between the Isar and Lech River catchments (Figure 2). The overall PCoA pattern was consistent with the Neighbor‐Net and Bayesian analyses, as no grouping of populations according to river catchment was observed. Most natural populations overlapped in the ordination space, except for population AM1, which appeared more distinct from the others.

**FIGURE 3:**
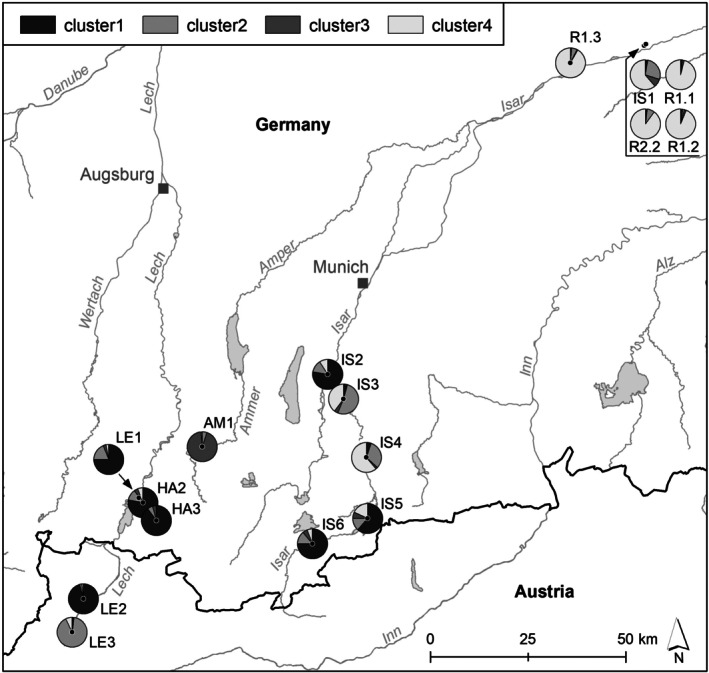
Genetic clustering of *Myricaria germanica* populations based on STRUCTURE analysis (*K* = 4). Cluster 1 dominates in populations from the upper Isar (IS2, IS5, IS6), Lech and Halblech Rivers (LE1, LE2, HA2, HA3), while LE3 and IS3 show higher proportions of cluster 2. The Ammer River population segregates clearly in cluster 3. All restored populations are assigned to the same genetic cluster as the ancestral population IS1 (cluster 4). Population IS4 also exhibits its highest membership proportion in cluster 4.

The number of clusters in STRUCTURE was set to four, as the mean logarithm of the probability of the data [Ln *p*(*X*|*K*)] approached a plateau at *K* = 4 (Figure S2) and further increases in *K* provided little additional information. Cluster 1 dominated in the upper Isar River (IS5, IS6), as well as IS2, and in the Lech and Halblech Rivers (LE1, LE2, HA2, HA3), except for populations LE3 and IS3, where Cluster 2 was most prevalent. A clear segregation of the Ammer River population (AM1) was observed in Cluster 3. The restored populations, together with the ancestral population IS1, were assigned to Cluster 4, highlighting their genetic affinity; notably, population IS4 also showed the highest proportion in this cluster. Overall, populations from the two river catchments were not separated into distinct clusters but were intermixed (Figure 3). This pattern is consistent with the Neighbor‐Net and PCoA analyses, in contrast to solutions with *K* = 2 or *K* = 3, which only partially explained the population structure (Figure S3).

Assignment tests detected four groups of individuals (Table S4). The first group consisted of individuals correctly assigned to their population of origin. In some populations (IS2, IS3, IS4, AM, HL2, HL3, LE3), 60%–100% of individuals were correctly assigned, whereas in others (IS1, IS5, IS6, LE1, LE2) only 7%–20% originated from their own site. The second group comprised mismatched allocations, that is, individuals assigned to other populations. Of these, 13%–40% (mean 23.5%) from IS1, IS2, IS5, and LE1 were allocated to upstream populations, while 6%–19% (mean 8.8%) from IS3, IS4, IS5, IS6, and LE2 were allocated downstream. The third group (7%–20%, mean 12%) included individuals allocated to populations outside their catchment (IS1, IS4, IS6, LE1, LE3, HL2). Finally, the fourth group (0%–81%, mean 33%) could not be assigned to any of the studied populations. All calculations were performed using MLDs of 0.5.

## 4 Discussion

### 4.1 Genetic Diversity of the Natural Populations

The mean genetic diversity observed within the natural populations corresponds to values reported for plant species with similar life history traits, as shown in the large‐scale comparative study by Reisch and Bernhardt‐Römermann (2014). Genetic diversity in the Asian congener *Myricaria laxiflora* showed greater variation, but mean values remained comparable (Liu et al. 2006). Previous research has suggested that population size may predict genetic diversity within populations (Leimu et al. 2006), with theory and empirical evidence frequently supporting a positive correlation between census size and genetic diversity (Frankham 1996). In contrast, riparian species inhabiting dynamic floodplain habitats, including *M. germanica* , have often been reported to lack such a correlation (Tero et al. 2003; Prentis et al. 2005; Werth and Scheidegger 2014; Fink et al. 2022). In our study, a significant correlation was detected in only one of five indices of genetic diversity (PPL). This outcome may reflect the high dispersal potential of *M. germanica* , its considerable seed output (Fink et al. 2017), and its capacity for selfing even in large populations. In extreme cases, large populations may descend from a single individual (Fink et al. 2022). Remarkably, the smallest natural population in our study, IS1 in the Isar catchment, consisting of mature to senile individuals, exhibited unexpectedly high genetic diversity, with the highest value for Nei's gene diversity (*H*). This population subsequently served as the seed source for the restored populations analyzed in our study.

### 4.2 Genetic Differentiation and Structure in the Natural Populations

Genetic differentiation among natural populations of *M. germanica* was comparable to levels reported for rare species and taxa with mixed mating systems (Nybom and Bartish 2000; Reisch and Bernhardt‐Römermann 2014). In the Asian congener *M. laxiflora* , even higher differentiation among populations has been observed (Liu et al. 2006). Genetic differentiation between adjacent populations along the Isar River varied considerably (*Φ*_PT_ = 0.00–0.33). Many natural populations along the Isar have disappeared due to habitat destruction, creating gaps that eliminate “stepping stones” which previously facilitated gene flow along the river corridor (Pollux et al. 2009; Werth and Scheidegger 2014). A similar pattern was detected in more extensive natural habitats, such as the Tagliamento River, where high isolation among *M. germanica* populations was attributed to unsuitable drought conditions prevailing along long stretches of the river course (Werth et al. 2014).

Genetic differentiation between adjacent populations along the Isar River varied considerably (*Φ*_PT_ = 0.00–0.33). The loss of many natural populations through habitat destruction has disrupted “stepping stones” that once facilitated gene flow along the river corridor (Pollux et al. 2009; Werth and Scheidegger 2014). A similar pattern was observed in the more extensive habitats of the Tagliamento River, where pronounced isolation among *M. germanica* populations was attributed to unsuitable drought conditions prevailing along extended stretches of the river course (Werth et al. 2014).

In contrast, genetic differentiation between the Isar and Lech catchments was surprisingly low, suggesting dispersal events across the two river systems (Fer and Hroudova 2009). Shared ancestry may also have contributed to this pattern. Furthermore, we found no clear pattern of isolation by distance (IBD), i.e., no correlation between genetic and geographical distances among the five populations along the Lech River; for the Isar River, this correlation was only marginally significant. Such a lack of association has frequently been reported for riparian and aquatic species, including *M. germanica* (Prentis et al. 2005; Pollux et al. 2009; Honnay et al. 2010; Fink et al. 2022). In our study, the missing overall IBD pattern likely reflects long‐distance dispersal events within a (previously) dynamic riverine system, as outlined in the preceding discussion.

In contrast to the AMOVA results, principal coordinate analysis and Neighbor Net indicated that most natural populations were not clearly separated, reflecting dispersal events and gene flow among them and consistent with subsequent analyses. This perceived discrepancy can be explained by methodological differences in how genetic structure is represented: While PCoA reveals substantial overlap among populations, suggesting weak spatial separation and ongoing gene flow, AMOVA indicates a relatively high level of genetic differentiation (∼30%) among populations. This contrast likely reflects low within‐population genetic diversity, which enhances the relative weight of between‐population differentiation in the AMOVA framework. In such cases, even modest differences among populations may appear more pronounced when within‐population diversity is limited. Thus, the results are not contradictory but complementary, illuminating different aspects of the genetic structure in the studied populations.

In line with this, Bayesian cluster analysis (STRUCTURE) and assignment tests revealed consistent patterns across the studied populations. STRUCTURE identified four genetic clusters, with Cluster 3 corresponding to the isolated population AM1 on the Ammer River. The remaining clusters encompassed both the Isar and Lech catchments, indicating that genetic structuring was not confined to individual river systems. This pattern suggests historical or ongoing gene flow between catchments. Assignment tests supported this interpretation, indicating several dispersal events between the Isar and Lech rivers. Similar inter‐catchment connectivity has been reported for other riparian and aquatic species (Ngeve et al. 2017; Fer and Hroudova 2009) and for *M. germanica* (Werth and Scheidegger 2014) with dispersal likely facilitated by animals or human activity.

Assignment tests revealed high proportions of individuals originating from their respective sampling sites, particularly in populations along the middle course of the Isar, the Halblech and Ammer Rivers, and LE3 on the upper Lech River. These results suggest limited seed dispersal and strong local recruitment, consistent with previous findings on wind dispersal in *M. germanica* , where the dispersal kernel typically spans 10–25 m from the fruiting shrub (Fink et al. 2017). In contrast, five populations—mostly located in dynamic or downstream segments—showed elevated proportions of non‐local genotypes. Together, the STRUCTURE and assignment results point to inter‐population input of diaspores and downstream accumulation, likely driven by hydrological connectivity and landscape dynamics.

In this context, the use of mixed seed sources in future reintroductions may appear promising, as they resemble natural processes and could hypothetically enhance genetic diversity. However, this approach requires careful consideration. Signals of pronounced genetic differentiation between populations highlight the potential risk of outbreeding depression, which may only become apparent in later generations. This underscores the need for long‐term monitoring in reintroduction projects using multiple seed sources. Moreover, evidence supporting the long‐term sustainability of such strategies without adverse genetic effects remains scarce and, to our knowledge, is lacking for *M. germanica* .

### 4.3 Genetic Diversity and Differentiation of Restored Populations

Within the restored populations, genetic diversity was slightly higher than in the natural Isar populations, although the difference was not significant. Comparable studies likewise reported restored populations to exhibit similar or marginally increased diversity (Betz et al. 2013; Millar, Anthony, et al. 2019; Millar, Coates, et al. 2019). On the contrary, restored populations may also exhibit reduced genetic diversity compared to their sources (Liu et al. 2008; Zucchi et al. 2018). General conclusions are therefore difficult, as restoration effects depend on species traits and project‐specific circumstances. In the case of *M. germanica* , however, restoration can be considered successful since no genetic depauperation was observed in the restored populations.

As expected, the highest number of private fragments occurred in the now isolated Ammer River population, a tributary of the Isar River. In contrast, restored populations lacked private fragments, likely reflecting sampling processes comparable to natural colonization events. Private alleles may thus serve as predictors of long‐term population persistence without severe bottlenecks (Ekrtová et al. 2012; Záveská et al. 2021). Rare fragments (≤ 5%) were detected in both natural and restored populations; notably, those retained in restored populations were shared across populations from all river catchments studied.

A spontaneous offspring population (R2.2) emerged near R1.2, displaying apparently higher genetic diversity than its parental population. This increase may reflect pollen flow from the neighboring, genetically variable population IS1.

Between the natural seed‐source and the group of restored populations, 8% of the variation was explained, and 4% among populations within groups, but neither was statistically significant. The overall *Φ*_PT_ value of 0.12, although proportionally small, was significant, indicating a detectable but weak genetic differentiation. Thus, restored populations retain most variation within populations, yet are not entirely homogeneous with their source. Previous projects that carefully collected seeds from donor populations reported only negligible genetic differences between natural and restored populations (Pierson et al. 2007; Ritchie and Krauss 2012; Betz et al. 2013; Millar, Anthony, et al. 2019). Similarly, in our project, the entire source population was sampled each year during seed collection. However, not all shrubs flowered and fruited annually, so the effective seed pool varied from year to year. This irregular reproductive output of the senescent source population IS1 was directly reflected in the plant material produced and used for restoration.

In this context, it is important to note that the Neighbor‐Net analysis confirmed genetic similarity between the natural seed‐source IS1 and the restored populations R1.1, R1.2, R1.3, as well as the offspring population R2.2. Notably, R2.2, derived from R1.2, showed surprisingly high similarity with IS1, supporting the assumption of pollen flow between IS1 and R1.2. This underscores the importance of founding restored populations near natural source stands (compare also Wagner and Woellner 2024), particularly in long‐lived species (Pierson et al. 2007), since spatial proximity of genetically valuable populations can mitigate founder or bottleneck effects in offspring populations.

## 5 Conclusions and Implications for Future Restoration Efforts

- Restored populations showed diversity levels comparable to natural populations, suggesting that restoration efforts were largely successful in maintaining within‐population variation. The overall *Φ*_PT_ value of 0.12, though modest, was significant, indicating weak but detectable genetic differentiation. Thus, while restored populations retained most of their variation within populations, they were not entirely homogeneous with their source. This slight differentiation was likely driven by uneven seed sampling across multiple years, as senile shrubs in the source population did not fruit consistently. Such irregularity may have contributed to minor shifts among restored sites. Overall, restoration outcomes were encouraging, while at the same time emphasizing the need for consistent and representative seed sourcing in future projects.
- In natural populations, genetic diversity metrics (e.g., Ne, H, SI, DW) varied among populations within each river system, but overall levels were comparable between the Isar and Lech Rivers. The only notable exception was the percentage of polymorphic loci (PPL), which was higher in the Lech. Despite this difference, the general pattern points to similar levels of genetic diversity across both river systems. Genetic differentiation among natural populations was moderate to high, with ΦPT values indicating significant structure within the river systems. However, hierarchical AMOVA showed that only 4% of the total genetic variation was attributable to differences between the river catchments. This surprisingly low value suggests ongoing or historical gene flow between the systems, likely facilitated by waterfowl dispersal or anthropogenic seed movement. In line with this interpretation, further analyses (PCoA, NeighborNet, STRUCTURE) revealed low levels of overall structuring, reflecting both past and present dispersal events within and between the river systems.
- Our study revealed no consistent pattern of genetic differentiation among natural populations along the Isar and Lech Rivers, reflecting the historically, and in some stretches still, dynamic character of alpine river systems. Furthermore, assignment tests indicate that in largely natural rivers, long‐distance gene flow among *M. germanica* populations is common. In this context, mixed seed sources in future reintroductions may appear beneficial, as they could enhance genetic diversity. Yet caution is required: pronounced genetic differentiation carries the risk of outbreeding depression and reduced fitness, which may only become evident in later generations. This concern should be explicitly addressed in future studies within the restoration discourse.

At the same time, our results indicate that restoration can effectively rely on a single genetically variable source population to sustain local diversity. In doing so, it safeguards genetic integrity and supports the long‐term persistence of this iconic riverine species in the face of environmental change.

## Author Contributions

**Katerina Iberl:** conceptualization (equal), data curation (equal), formal analysis (lead), investigation (lead), methodology (equal), visualization (lead), writing – original draft (lead), writing – review and editing (lead). **Christoph Reisch:** conceptualization (equal), data curation (equal), formal analysis (supporting), investigation (supporting), methodology (equal), visualization (supporting), writing – original draft (supporting), writing – review and editing (supporting).

## Conflicts of Interest

The authors declare no conflicts of interest.

## Supporting information


**Table S1:** Private fragments within natural and restored populations.
**Table S2:**. Rare fragments (i.e., frequency lower than 5%) present in the natural and restored populations.
**Table S3:** Pairwise PhiPT values between all populations of *Myricaria germanica* calculated employing AMOVA.
**Table S4:** Assignment test results for all individuals of *Myricaria germanica* sampled from natural populations. The analysis reveals individual affiliations across the studied populations.

**Figure S1:** Pearson correlation coefficient showed a positive, moderately strong (*R* = 0.57) marginally significant (*p* = 0.055) association between the variables. The x‐axis corresponds to the log transformed population size; the y‐axis describes the genetic diversity (PPL).
**Figure S2:** Mean posterior probability plot of STRUCTURE (STRUCTURE, Pritchard et al. 2000) over 20 runs for each value of *K*. The graph shows the mean ln Pr (*X*|*K*) over 20 iterations and standard deviation for each value of *K*.
**Figure S3:** Results of the Bayesian cluster analysis for all individuals of *Myricaria germanica*. Shown are solutions in which the 16 study populations were assigned to two (a), three (b), and four (c) clusters, respectively. Each bar represents one individual. In none of the solutions did population clustering correspond to river catchments. (a) *K* = 2: Bayesian cluster analysis of 16 populations of *Myricaria germanica* with assignment to two clusters. (b) *K* = 3: Bayesian cluster analysis of 16 populations of *Myricaria germanica* with assignment to three clusters. (c) *K* = 4: Bayesian cluster analysis of 16 populations of *Myricaria germanica* with assignment to four clusters.

## Data Availability

All relevant data are given in the article or in the article Tables S1–S4 and Figures S1–S3.
