# Supplementary material for: Reintroduction Without Genetic Bottlenecks: Preserving Diversity in Restored Populations of the Critically Endangered Riverine Shrub Myricaria germanica
Source: Ecol Evol. 2026 Jan 11;16(1):e72879. doi: 10.1002/ece3.72879 (PMC12793041; doi:10.1002/ece3.72879)
Supplement: Supplementary file 1 — Table S1: Private fragments within natural and restored populations. Table S2:. Rare fragments (i.e., frequency lower than 5%) present in the natural and restored populations. Table S3: Pairwise PhiPT values between all populations of Myricaria germanica calculated employing AMOVA. Table S4: Assignment test results for all individuals of Myricaria germanica sampled from natural populations. The analysis reveals individual affiliations across the studied populations. [file ECE3-16-e72879-s001.docx]

**Appendix / Tables**

**Table A1.** Private fragments within natural and restored populations.

| **Population** | **F_004** | **F_049** | **F_051** | **F_144** | **F_164** | **Sum per population** |
| --- | --- | --- | --- | --- | --- | --- |
| **IS6** | 0 | 1 | 0 | 0 | 0 | **1** |
| **AM1** | 1 | 0 | 1 | 1 | 0 | **3** |
| **LE2** | 0 | 0 | 0 | 0 | 1 | **1** |

The first column lists the population names, while the top row indicates the fragment names. The final column presents the total number of private fragments found within each population

**Table A2**. Rare fragments (i.e. frequency lower than 5%) present in the natural and restored populations.

| **Population** | **F_040** | **F_041** | **F_045** | **F_047** | **Sum per population** |
| --- | --- | --- | --- | --- | --- |
| **IS2** | 0 | 0 | 1 | 0 | **1** |
| **IS5** | 1 | 0 | 0 | 0 | **1** |
| **AM1** | 0 | 0 | 0 | 1 | **1** |
| **LE2** | 0 | 1 | 0 | 0 | **1** |
| **HA1** | 0 | 0 | 1 | 0 | **1** |
| **HA2** | 0 | 0 | 1 | 0 | **1** |
| **R1.1** | 0 | 0 | 0 | 1 | **1** |
| **R1.2** | 1 | 0 | 1 | 0 | **2** |
| **R2.2** | 0 | 1 | 0 | 0 | **1** |

The first column lists the population names, while the top row indicates the fragment names. The final column presents the total number of rare fragments found within each population.

**Table A3.** Pairwise PhiPT values between all populations of *Myricaria germanica* calculated employing AMOVA*.*

|  | IS1 | IS2 | IS3 | IS4 | IS5 | IS6 | AM | LE1 | HL2 | HL3 | LE2 | LE3 | R1.1 | R1.2 | R1.3 | R2.2 |
| --- | --- | --- | --- | --- | --- | --- | --- | --- | --- | --- | --- | --- | --- | --- | --- | --- |
| IS1 | 0.000 | 0.001 | 0.032 | 0.001 | 0.003 | 0.001 | 0.001 | 0.002 | 0.001 | 0.001 | 0.001 | 0.002 | 0.004 | 0.026 | **0.312** | **0.300** |
| IS2 | 0.33 | 0.000 | 0.001 | 0.001 | 0.001 | 0.001 | 0.001 | 0.001 | 0.001 | 0.001 | 0.001 | 0.001 | 0.001 | 0.001 | 0.001 | 0.001 |
| IS3 | 0.09 | 0.28 | 0.000 | 0.001 | 0.001 | 0.001 | 0.001 | 0.001 | 0.001 | 0.001 | 0.001 | 0.001 | 0.001 | 0.001 | 0.001 | 0.001 |
| IS4 | 0.22 | 0.32 | 0.13 | 0.000 | 0.001 | 0.001 | 0.001 | 0.001 | 0.001 | 0.001 | 0.001 | 0.001 | 0.001 | 0.001 | 0.001 | 0.001 |
| IS5 | 0.18 | 0.24 | 0.17 | 0.20 | 0.000 | **0.140** | 0.001 | 0.001 | 0.001 | 0.001 | 0.001 | 0.001 | 0.001 | 0.001 | 0.001 | 0.001 |
| IS6 | 0.19 | 0.24 | 0.20 | 0.23 | 0.02 | 0.000 | 0.001 | 0.001 | 0.001 | 0.001 | 0.001 | 0.001 | 0.001 | 0.001 | 0.001 | 0.001 |
| AM | 0.53 | 0.59 | 0.48 | 0.59 | 0.47 | 0.51 | 0.000 | 0.001 | 0.001 | 0.001 | 0.001 | 0.001 | 0.001 | 0.001 | 0.001 | 0.001 |
| LE1 | 0.21 | 0.16 | 0.22 | 0.22 | 0.10 | 0.10 | 0.43 | 0.000 | 0.001 | 0.001 | **0.100** | 0.001 | 0.001 | 0.001 | 0.001 | 0.001 |
| HL2 | 0.34 | 0.30 | 0.31 | 0.30 | 0.27 | 0.24 | 0.53 | 0.11 | 0.000 | 0.001 | 0.001 | 0.001 | 0.001 | 0.001 | 0.001 | 0.001 |
| HL3 | 0.45 | 0.32 | 0.39 | 0.49 | 0.34 | 0.37 | 0.55 | 0.23 | 0.27 | 0.000 | 0.001 | 0.001 | 0.001 | 0.001 | 0.001 | 0.001 |
| LE2 | 0.37 | 0.23 | 0.33 | 0.36 | 0.15 | 0.15 | 0.59 | 0.02 | 0.23 | 0.33 | 0.000 | 0.001 | 0.001 | 0.001 | 0.001 | 0.001 |
| LE3 | 0.26 | 0.39 | 0.22 | 0.21 | 0.31 | 0.32 | 0.62 | 0.25 | 0.39 | 0.53 | 0.43 | 0.000 | 0.001 | 0.001 | 0.001 | 0.001 |
| R1.1 | 0.16 | 0.37 | 0.24 | 0.30 | 0.29 | 0.33 | 0.62 | 0.31 | 0.47 | 0.54 | 0.44 | 0.39 | 0.000 | 0.101 | 0.038 | 0.001 |
| R1.2 | 0.13 | 0.39 | 0.22 | 0.29 | 0.29 | 0.34 | 0.61 | 0.32 | 0.48 | 0.55 | 0.44 | 0.39 | 0.04 | 0.000 | **0.131** | 0.008 |
| R1.3 | 0.01 | 0.30 | 0.18 | 0.20 | 0.21 | 0.26 | 0.56 | 0.26 | 0.39 | 0.47 | 0.38 | 0.31 | 0.06 | 0.04 | 0.000 | **0.312** |
| R2.2 | 0.02 | 0.28 | 0.19 | 0.27 | 0.23 | 0.27 | 0.55 | 0.28 | 0.42 | 0.47 | 0.37 | 0.31 | 0.13 | 0.08 | 0.01 | 0.000 |

Below the diagonal: pairwise PhiPT values; above the diagonal: probability values based on 999 permutations. Non-significant values are highlighted in bold

**Table A4.** Assignment test results for all individuals of *Myricaria germanica* sampled from natural populations. The analysis reveals individual affiliations across the studied populations.

|  | To | | | | | | | | | | | |
| --- | --- | --- | --- | --- | --- | --- | --- | --- | --- | --- | --- | --- |
| From | IS1 | IS2 | IS3 | IS4 | IS5 | IS6 | AM | LE1 | HL2 | HL3 | LE2 | LE3 |
| IS1 | **1, 1, 1** | 0, 0, 0 | 3, 2, 1 | 0, 0, 0 | 0, 0, 0 | 1, 0, 0 | 0, 0, 0 | 0, 0, 0 | 0, 0, 0 | 0, 0, 0 | 0, 0, 0 | 0, 0, 0 |
| IS2 | 0, 0, 0 | **12, 11, 11** | 0, 0, 0 | 0, 0, 0 | 0, 0, 0 | 1, 1, 1 | 0, 0, 0 | 0, 0, 0 | 1, 1, 1 | 0, 0, 0 | 0, 0, 0 | 0, 0, 0 |
| IS3 | 0, 0, 0 | 2, 2, 2 | **11, 10, 10** | 1, 1, 0 | 2, 1, 1 | 0, 0, 0 | 0, 0, 0 | 1, 0, 0 | 0, 0, 0 | 0, 0, 0 | 0, 0, 0 | 1, 1, 1 |
| IS4 | 0, 0, 0 | 0, 0, 0 | 1, 1, 0 | **11, 11, 11** | 0, 0, 0 | 0, 0, 0 | 0, 0, 0 | 1, 1, 1 | 2, 0, 0 | 0, 0, 0 | 0, 0, 0 | 0, 0, 0 |
| IS5 | 0, 0, 0 | 0, 0, 0 | 0, 0, 0 | 1, 0, 0 | **7, 1, 1** | 6, 3, 2 | 0, 0, 0 | 0, 0, 0 | 0, 0, 0 | 0, 0, 0 | 0, 0, 0 | 0, 0, 0 |
| IS6 | 2, 1, 1 | 0, 0, 0 | 0, 0, 0 | 0, 0, 0 | 5, 4, 3 | **6, 4, 3** | 0, 0, 0 | 0, 0, 0 | 0, 0, 0 | 0, 0, 0 | 0, 0, 0 | 0, 0, 0 |
| AM | 1, 0, 0 | 0, 0, 0 | 0, 0, 0 | 0, 0, 0 | 0, 0, 0 | 1, 1, 1 | **13, 13, 13** | 0, 0, 0 | 0, 0, 0 | 0, 0, 0 | 0, 0, 0 | 0, 0, 0 |
| LE1 | 0, 0, 0 | 0, 0, 0 | 0, 0, 0 | 0, 0, 0 | 0, 0, 0 | 0, 0, 0 | 0, 0, 0 | **6, 3, 2** | 1, 0, 0 | 0, 0, 0 | 3, 1, 1 | 0, 0, 0 |
| HL2 | 0, 0, 0 | 0, 0, 0 | 0, 0, 0 | 0, 0, 0 | 0, 0, 0 | 0, 0, 0 | 0, 0, 0 | 0, 0, 0 | **10, 10, 9** | 1, 1, 0 | 1, 0, 0 | 0, 0, 0 |
| HL3 | 0, 0, 0 | 0, 0, 0 | 0, 0, 0 | 0, 0, 0 | 0, 0, 0 | 0, 0, 0 | 0, 0, 0 | 0, 0, 0 | 0, 0, 0 | **14, 13, 13** | 0, 0, 0 | 0, 0, 0 |
| LE2 | 0, 0, 0 | 1, 1, 0 | 0, 0, 0 | 0, 0, 0 | 0, 0, 0 | 0, 0, 0 | 0, 0, 0 | 6, 5, 5 | 1, 0, 0 | 0, 0, 0 | **12, 2, 1** | 0, 0, 0 |
| LE3 | 1, 1, 1 | 0, 0, 0 | 1, 0, 0 | 3, 3, 3 | 0, 0, 0 | 1, 1, 1 | 0, 0, 0 | 1, 1, 1 | 0, 0, 0 | 0, 0, 0 | 0, 0, 0 | **13, 11, 10** |
|  | | | | | | | | | | | | |
| **Summary** | | | | | | | | | | | | |
| n | 5 | 15 | 16 | 16 | 14 | 16 | 13 | 15 | 15 | 15 | 16 | 14 |
| CA (n) | 1, 1, 1 | 12, 11, 11 | 11, 10, 10 | 11, 11, 11 | 7, 1, 1 | 6, 4, 3 | 13, 13, 13 | 6, 3, 2 | 10, 10, 9 | 14, 13, 13 | 12, 2, 1 | 13, 11, 10 |
| MA (n) | 4, 2, 2 | 3, 2, 2 | 5, 3, 1 | 5, 5, 4 | 7, 5, 4 | 10, 6, 5 | 0, 0, 0 | 9, 7, 7 | 5, 1, 1 | 1, 1, 0 | 4, 1, 1 | 1, 1, 1 |
| NA (n) | 0, 2, 2 | 0, 1, 2 | 0, 3, 5 | 0, 0, 1 | 0, 8, 9 | 0, 6, 8 | 0, 0, 0 | 0, 5, 6 | 0, 4, 5 | 0, 1, 2 | 0, 11, 13 | 0, 2, 3 |

Results of individual assignment tests for 170 *Myricaria germanica* individuals from natural populations, based on three thresholds of minimal log-likelihood difference (MLD): 0, 0.3, and 0.5. Bold numerals indicate the number of correctly assigned individuals per population. CA = correctly allocated; MA = mismatched allocation; NA = non-allocated individuals. For methodological details, see Materials and Methods.
